# Evaluation of Explainable Artificial Intelligence in IoT Intrusion Detection Systems Under DeepFool Adversarial Conditions

**DOI:** 10.3390/s26102924

**Published:** 2026-05-07

**Authors:** Jorge Munilla, Rana M. Khammas

**Affiliations:** 1 Department of Communication Engineering, E.T.S.I. de Telecomunicación, Universidad de Málaga, Campus de Teatinos, 29190 Málaga, Spain; rana.m.khammas@uma.es; 2 Scientific Research Commission, Baghdad 10070, Iraq

**Keywords:** intrusion detection systems, explainable artificial intelligence, SHAP, LIME, IoT, sparsity, completeness, robustness, DeepFool algorithm

## Abstract

As IoT systems complexity grows, transparent and trustworthy machine-learning intrusion detection systems are crucial. Post hoc explainable AI methods, such as SHAP and LIME, are the most widely used ways to explain how models work, but the degree to which these methods are robust to adversarial conditioning is understudied. In this paper, we propose to create a unified system of evaluating explanation fidelity by using three metrics: sparsity, completeness, and robustness based on minimally distorting DeepFool input perturbations. Our study benchmarks SHAP and LIME across three datasets (BoT-IoT, Edge-IIoT, and N-BaIoT) using four classifiers: CNN, DNN, LSTM, and RF. Our results demonstrate a consistent trade-off: SHAP achieves stronger feature alignment and higher completeness under attack, whereas LIME exhibits greater rank stability in terms of top-k feature overlap. However, LIME also produces more spurious attributions and offers less explanatory power than SHAP, especially in the presence of synthetic features. Our findings reveal that high model accuracy does not guarantee that the provided explanation is also high-fidelity. This investigation highlights the necessity for robustness-aware XAI in cybersecurity and provides reproducible parameters to guide the adoption of XAI in adversarial environments.

## 1. Introduction

The proliferation of Internet of Things (IoT) botnets [1] has changed the security landscape for Internet-attached devices. IoT botnets are a growing concern for the security of homes, businesses, and critical infrastructure. A botnet is defined as a collection of Internet-enabled devices that are controlled by a single entity (botmaster). The botmaster is able to use the botnet for malicious purposes, including distributed denial of service attacks, spamming, and cryptocurrency mining efforts. Due to the number of devices connected to the Internet, the size of botnets of IoT devices can be larger compared to traditional botnets, meaning they are a larger threat to Internet security [2]. Detecting IoT botnets is challenging because they are highly distributed and not confined to any single type of device [1]. IoT botnets exist in such a way that current detection methodologies, like signature-based detection and anomaly-based detection, are not effective. Botmasters can vary the specific behaviors of their IoT botnet quickly to evade detection. Here, the promise of machine learning (ML) comes into play as a tactic to detect IoT botnets, given the size of data that can be analyzed and the complexity and patterns in the data itself that are unlikely to be recognized by humans [3,4,5,6].

Despite the myriad impressive results delivered by many ML methods in the cybersecurity space, there is serious concern due to the intrinsic lack of interpretability of ML models, which means security experts may struggle to trust what an ML model produces because they do not fully understand how a model arrives at a specific decision or classification. Trust is a significant hurdle in intrusion detection systems and many other mission-critical systems. Consequently, a number of novel approaches have been developed in recent years to enhance the explainability of ML models, thereby facilitating more interpretable outputs. This idea is referred to as explainable artificial intelligence in the literature [6]. The assimilation of XAI methods has created a new focus in cybersecurity research to develop and include additional layers of explainability for humans in the loop [7,8]. Several works have considered XAI methods to be useful for IoT botnet detection systems [9,10]. Some survey papers describe current open questions and future research directions [11,12,13,14]. Collectively, these works highlight open issues around standardized evaluation and reproducibility, robustness of explanations under adversarial perturbations and distribution shift, resource-aware deployment on constrained IoT/edge devices, bridging faithfulness versus plausibility, operational integration with analysts, and privacy-security risks arising from explanations themselves. In this work, however, SHAP and LIME are used as post hoc analysis tools within an offline evaluation setting, rather than as explanation mechanisms intended for direct execution on resource-constrained IoT end devices.

Typically, many learned models, like convolutional neural networks, can be treated as “black boxes”, which do not allow verification of the means of decision [15]. Although the purpose of explainable AI is to gain insights into “how” ML models use features, for example, when one is detecting potential IoT botnets, an XAI method can reveal insights into which network features are important in detecting the presence of malicious activity on an IoT device. The information produced by any XAI method is referred to as an “explanation”. Explanations are useful in improving understanding of the models and, along with that, create trust in the decisions made. Model creators and ML scientists can utilize explanations to enhance the model’s performance quality. Furthermore, XAI methodologies can assist in improving a model regardless of whether the explanation is global or local in nature; while global XAI aims to describe the overall behavior of the model, local XAI focuses on explaining individual predictions. It is also relevant to note that in an XAI model, one can achieve explanation using intrinsic (model-specific) XAI, which means that explainability is part of the learning procedure during training of the model (and is not transferable), or via a post hoc (model-agnostic) XAI approach. Post hoc XAI methods are model-agnostic, meaning they do not depend on any specific trained or pre-trained ML model architecture [16].

The rise of XAI techniques, such as Shapley Additive exPlanations (SHAP) [17] and Local Interpretable Model-agnostic Explanations (LIME) [6], seems to emphasize the desperate need for transparent and trustworthy explanations in cybersecurity. Although XAI tools are projected to become a staple of network security, supporting analysts in making decisions more rapidly and accurately, there remains a significant challenge in validating and evaluating these interpreters in real-world Intrusion Detection System settings. Establishing reliable validation procedures and defining security metrics relevant to usability are particularly difficult, as no standardized methods currently exist for assessing the quality and effectiveness of XAI in practical deployments. The extent of validation defines the usability of XAI-based tools but may also hinder the use of AI-based forms of XAI altogether. If there is no standard way to measure and model the utility of the evaluations, it diminishes the usefulness of the security professionals who rely on them for decisions leading to potentially dire consequences [18].

Prior research [19,20,21,22] has established that post hoc explanation methods, such as SHAP and LIME, are susceptible to distortion through adversarial model training, leading to compromised feature attribution and misleading explanations. These previous studies primarily exposed weaknesses in XAI by constructing biased or adversarial models for analysis, but they largely evaluated these models using synthetic attack scenarios. They did not assess explanation methods’ performance against realistic adversarial perturbations, such as those enforced by DeepFool. DeepFool is an adversarial attack method that can minimize perturb inputs to manipulate predictions from the model and modify the input without a loss of the original data’s meaning. It is highly efficient and typically generates adversarial examples that remain very close to the original input. We refer the reader to the original work for a more detailed explanation of the mathematical formulation underlying its iterative procedure [23].

In this paper, we propose a framework for evaluating XAI methods for network intrusion detection systems using various metrics, such as sparsity, completeness, and robustness, which are defined as follows:

Sparsity. Sparsity refers to the extent to which an explanation concentrates its importance on a limited subset of features while assigning relatively low importance to the remaining features. A sparse explanation is therefore characterized by a concentrated attribution pattern, which may help security analysts focus on a smaller set of influential traffic attributes. To evaluate this behavior, we analyze the cumulative distribution of normalized feature-importance scores across multiple thresholds.

Completeness. Completeness refers to the extent to which an XAI method highlights features that align with perturbation-sensitive input dimensions under adversarial conditions. A lower completeness score indicates that the explanation may fail to capture input features associated with decision-sensitive changes, which may reduce its usefulness in adversarial intrusion detection settings.

Robustness. Robustness refers to the extent to which an XAI method preserves its explanatory feature rankings when the input is subjected to small adversarial perturbations. A robust explanation method should maintain a stable attribution structure before and after perturbation, even when the perturbation is generated by an attack such as DeepFool.

We explore the explainability of the learned model by applying the three evaluation criteria defined above, using two popular black-box XAI methods introduced earlier: LIME and SHAP. Both are well-known and regularly used as XAI methods in the network intrusion research domain [24,25,26,27]. LIME provides local explanations to understand how the input contributes to the decision. It builds upon a linear model, where it introduces perturbations to the sample and generates rules about how that model functions on that sample based on those perturbations. SHAP can be considered as both a local and a global explanation method. It evaluates the importance of each feature by determining the corresponding Shapley value for each feature based on the Shapley value concept from game theory [28]. If the output does not change when removing the feature with respect to the original decision of the AI model, then that feature is considered to be of minimal importance with respect to that AI model.

This paper describes the evaluation process step by step for the three evaluation metrics of sparsity, completeness, and robustness to produce our evaluation metrics and results using Python v3.12.7. The first dataset, BoT-IoT [29], was generated by simulating a realistic network environment at the UNSW Canberra Cyber Range Lab. It includes a mix of normal and botnet traffic. The second dataset, N-BaIoT [30], is associated with the issue of a lack of public botnet datasets available, especially for the IoT, introducing real traffic data collected from 9 commercial IoT devices that were authentically infected by Mirai and BASHLITE. The last dataset, Edge-IIoT [31], was collected from more than 10 types of devices, which included low-cost digital sensors. More details about these datasets are given later, in Section 3.1.

For each dataset, both XAI approaches, SHAP and LIME, will be evaluated across multiple black-box AI models, including Convolutional Neural Networks (CNNs), Deep Neural Networks (DNNs), Long Short-Term Memory networks (LSTMs), and Random Forests (RFs). We then present and analyze the results obtained from the three evaluation metrics computed for each XAI method. This framework aims to broaden the application of XAI within intrusion detection systems (IDS), thereby introducing greater realism to this research area and enabling further advancements.

In addition to the general goal of assessing sparsity, robustness, and completeness using DeepFool as the adversarial model, we highlight the following key aspects of this paper:

- A unified adversarial evaluation protocol based on DeepFool perturbations is developed to analyze the behavior of post hoc XAI methods near the model decision boundary.
- An empirical feature-alignment completeness measure is introduced to assess whether the top-k features identified by SHAP and LIME overlap with perturbation-sensitive input dimensions under adversarial perturbation.
- A robustness evaluation is conducted on the BoT-IoT, Edge-IIoT, and N-BaIoT datasets to compare the stability of SHAP and LIME explanations before and after DeepFool-based attacks.
- A top-k overlap-based robustness analysis is used to quantify the consistency of explanation features under adversarial perturbation across multiple samples and datasets.
- A sparsity analysis based on CTS curves and CTS-AUC is provided to compare how concentrated or diffuse the explanation scores are across different methods, models, and datasets.
- The proposed framework is applied across multiple intrusion detection datasets and classifiers to provide a comparative empirical analysis of explanation behavior under adversarial conditions.

The remainder of this paper is structured as follows: Section 2 provides a summary of related work. Section 3 details the methodology, including the datasets’ description, the AI and XAI models employed, and the evaluation procedure under DeepFool attacks. Section 4 presents and analyzes the experimental results, and finally, Section 5 concludes the paper.

## 2. Related Works

As the use of explainable artificial intelligence grows, important questions are arising regarding the reliability and robustness of the post hoc explanation techniques. Important issues in this regard were highlighted by [19], who critiqued popular explanation tools such as LIME and SHAP and demonstrated that these methods can be adversarially manipulated to produce misleading explanations while still yielding correct predictions. The authors noted that a classifier could be adversarially trained to yield the correct prediction while giving misleading explanations, thus being correct but not interpretable. In effect, this demonstrated a serious weakness in some of the existing XAI methods and prompted the research community to work toward more robust and faithful explanation methods.

While building on this weakness, one of the first studies that was conducted by [20] systematically reviewed XAI methods in a security-related context. The authors compared several saliency-based methods (e.g., LRP, Grad-CAM, and Integrated Gradients) and applied them to deep learning models for malware classification tasks. The findings from this study revealed that many explanation methods produced unstable and noisy attributions, particularly when inputs were changed very slightly, and the authors cautioned that explanations may often reflect model artifacts rather than any real rationale for the model’s decision. This challenge is especially concerning in consideration of the fact that XAI tools may fail at the very times they are needed most; i.e., in adversarial or other high-risk environments.

Building on this work, the TRUST framework proposed in [32] introduces an interpretable IDS for industrial IoT systems using LIME to promote transparency. Again, this failed to acknowledge [19] concerns about demonstrating the limitations of early-stage explainability frameworks in adversarial settings.

In 2022, these limitations were also addressed by [33], who proposed a framework that employed SHAP and RuleFit as add-ons on top of DNN for IoT IDS using the NSL-KDD and UNSW-NB15 datasets, while their approach enhanced interpretability by incorporating adversarial attacks, the framework was computationally expensive, highlighting the common trade-off between robustness and efficiency. [24] developed detection accuracy on large-scale IoT data by using SHAP with ensemble models (random forest/decision trees) to improve general interpretability. However, their approach was not applied to more recent and richer deep learning architectures. At the same time, LIME with ensemble classifiers was also used in [34] for improved interpretability and explained that they did not assess performance in high-velocity data streams, a key feature for real-time IDS systems.

In 2023, the XAI community began to shift toward evaluation-based work, including benchmark creation and quantitative evaluation. A dataset collection of comprehensive visual explanation studies (e.g., Gender-XAI, Nodule-XAI) was created by [35] and provided the evaluation of three methods: Grad-CAM, RISE, and ViT. Significant contributions were made [35] toward establishing a much-needed standard for evaluating XAI methods. However, they also identified challenges associated with using human-annotated data, such as the presence of biases, and raised concerns about whether models may have inadvertently exploited other datasets or spurious features. Such issues could undermine generalizability and fail to capture true task-specific similarities. In 2023, an interpretable fuzzy decision tree-based IDS for IoT networks was created by [36], which is an inherently interpretable (explainable) alternative, but the work was very preliminary and without an external validation study.

A real-time IDS using SHAP with a Random Forest model [37] was successfully implemented on the UNSW-NB15 dataset; however, the evaluation relied on this single dataset, limiting the validity and generalizability of their findings. In 2023, MEMC, a new XAI evaluation metric, was proposed by [38] and focused on healthcare XAI model interpretability. However, they also did not capture the computational costs specific to high dimensionality. Finally, completeness and correctness of explanation with LIME using multiple datasets were provided by [39], exposing dependencies on human feedback and classifier performance.

By 2024, the discussion and overall concern of evaluation metrics had a wide scope of maturity, and the Mean Opinion Score (MOS) [40] was validated as a user-driven measurement, showing only a weak correlation with other symptoms of automatic measures such as IAUC and DAUC, acknowledging the limitation of the subjective layer of example or explanation evaluation being unaddressed. Also in 2024, sMPRT and eMPRT frameworks were proposed by [41] as a structured way to engage in the evaluation of explanation mimicry and model complexity; while their extensibility is evident, the frameworks appear markedly complex and computationally expensive, both in terms of processing requirements and the interpretative challenges of inductive tasks, particularly given the extensive groundwork needed to establish evaluation metrics.

Wrapping up this line of research, ref. [21] introduced a comprehensive framework for evaluating black-box explanation techniques, particularly SHAP and LIME, in intrusion detection systems, considering both global and local perspectives across multiple IDS datasets. In a parallel direction, ref. [22] extended a similar evaluation philosophy to anomaly detection in autonomous driving systems and explicitly assessed SHAP and LIME across six evaluation metrics, including sparsity, completeness, and robustness. Together, these studies established an important foundation for examining explanation quality beyond predictive accuracy alone. However, they still leave open a critical question for adversarially exposed environments: how explanation fidelity should be assessed when explanations are stressed by minimal, decision-boundary-crossing perturbations rather than only by broad evaluation criteria.

Building on these foundations, our work provides a more adversarially grounded and application-specific evaluation of SHAP and LIME in IoT intrusion detection. More specifically, our methodology differs from [21,22] in the way completeness and robustness are operationalized. For completeness, instead of relying on a general explanation-quality criterion, we use a DeepFool-based feature-alignment formulation in which the original top-k features identified by SHAP or LIME are compared against the input dimensions perturbed along the minimal adversarial path, producing completeness curves ${\mathrm{COMP}}_{k}\left(\alpha \right)$. For robustness, rather than adopting a single generic robustness score, we evaluate explanation stability through three complementary tests: (i) a clean-versus-adversarial explanation comparison under DeepFool, (ii) a synthetic unrelated-feature test to reveal spurious attributions, and (iii) a top-k feature-overlap analysis with paired *t*-test and Wilcoxon signed-rank validation. In this sense, our study remains aligned with recent work while offering several concrete advances: a unified and reproducible evaluation framework across three IoT IDS benchmarks and four classifiers, a more fine-grained adversarial analysis of explanation behavior, and a clearer characterization of the trade-off between feature alignment and rank stability under adversarial conditions. Consequently, the contribution of this work is not limited to predictive performance but also lies in providing a more practically relevant empirical assessment of explanation behavior in adversarial IoT environments.

## 3. Methodology

**Figure 1:**
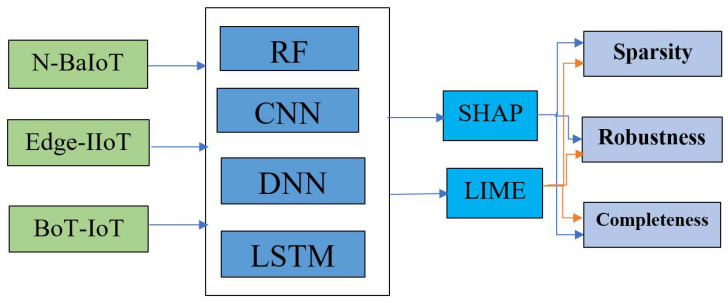
An overview of the XAI evaluation framework for network intrusion detection. Blue arrows represent SHAP explanations, while orange arrows represent LIME explanations.

This section presents the adversarial evaluation protocol adopted in this study to analyze the behavior of post hoc explanation methods across multiple intrusion detection datasets and machine learning models. As illustrated in Figure 1, the overall workflow comprises data preprocessing, classifier training, adversarial example generation using DeepFool, and explanation-based evaluation.

Rather than introducing a new explainer, the contribution of this section lies in establishing a unified empirical protocol for assessing explanation behavior under controlled adversarial perturbations. In contrast to prior studies that typically rely on isolated metrics or static post hoc comparisons, the proposed protocol integrates three complementary evaluation dimensions—sparsity, feature-alignment completeness, and robustness—within a single and consistent framework.

By combining progressive perturbation scaling, explanation–perturbation feature overlap analysis, and cross-model/cross-dataset validation, this protocol enables a more rigorous assessment of how explanation behavior changes under adversarial stress in intrusion detection settings.

### 3.1. Datasets Description

We employed three publicly available intrusion detection datasets:

BoT-IoT [29]: The BoT-IoT dataset was generated whilst using the Ostinato tool to generate network traffic data from a cloud server using virtual machines and Kali Linux machines running various services; these services included DNS, SSH, FTP, and HTTP. The Kali machines simulated the IoT devices in the experiment, and Node-RED was used as the programming tool to enhance the realistic behavior of each IoT device. The dataset includes 5 different IoT scenarios, with each scenario representing a different IoT device (weather station, smart refrigerator, motion-activated lights, remotely activated garbage door, and smart thermostat). The network traffic data that was collected contained several types of attacks that included UDP, TCP, OS fingerprint, service scan, HTTP, keylogging, and data exfiltration. The dataset is provided in two versions: a full version containing over 72 million records and a 10% version with approximately 3.6 million records. In this study, we adopted the publicly released 5% subset of the BoT-IoT dataset and its corresponding optimal feature set, following the benchmark setup reported in the original dataset study [29].

N-BaIoT [30]: For the N-BaIoT datasets, attacks are performed using C&C (Command and Control) servers, which are used by botmasters to control a network of infected IoT devices (bots). The C&C server is the microsystem that the botmaster uses to send commands to the bots and receive information the bots send back. The N-BaIoT dataset used Mirai and Gafgyt botnets to deliver malware and execute coordinated DDoS (Distributed Denial of Service) attacks. These botnets delivered malware to various IoT devices, including routers, cameras, and other smart devices, by exploiting various vulnerabilities. The N-BaIoT datasets include features recorded in this controlled testing environment. The datasets contain combined statistics of the raw network streams over five different time windows (100 ms, 500 ms, 1.5 s, 10 s, and 1 min), with 115 features obtained from the network traffic. The time windows are coded L0.01, L0.1, L1, L3, and L5, respectively. Each feature is assigned to five main categories of metrics, with the categories including: host-IP(H), host MAC&IP (MI), channel (HH), socket (HpHp), and network jitter (HH_Jit). The statistical values used are from the five higher-order metrics: the number of packets, the mean, and the variance of packet sizes. The channel and socket categories have additional values derived from packet size, radius, covariance, and magnitude, such as the correlation coefficient.

Edge-IIoT [31]: These datasets consist of data collected from more than 10 types of devices, including low-cost digital temperature and humidity sensors, pH meters, ultrasonic sensors, heart rate sensors, water-level detectors, soil moisture sensors, flame sensors, and similar equipment. In these databases, there are 14 different kinds of attacks with IoT and various IIoT protocols: MITM, fingerprinting, ransomware, uploading, sql injection, ddos_http, ddos_tcp, ddos_udp, ddos_icmp, password, port scanning, vulnerability scanner, backdoor, and XSS.

The preprocessing pipeline included label encoding, feature standardization, and dataset-specific class balancing. First, the target labels were converted into numerical form using LabelEncoder. This step was applied consistently across all datasets to ensure compatibility with the learning models. Next, feature scaling was performed using StandardScaler, where each feature was standardized to have zero mean and unit variance.

To address class imbalance, a balancing strategy was applied only when required by the dataset distribution. In this study, balancing was performed for the BoT-IoT dataset due to its pronounced class imbalance. Specifically, a two-stage random resampling strategy was employed, where RandomOverSampler was first used to increase the number of samples in minority classes, followed by RandomUnderSampler to reduce the majority classes and obtain a more balanced distribution. In contrast, Edge-IIoT and N-BaIoT were used without additional balancing, as their class distributions after preprocessing were considered sufficiently suitable for training and evaluation. We used 80% for the training stage and 20% for the testing stage. We did utilize all the features listed in Table 1 in our initial experiments. This comprehensive approach allowed us to fully leverage the datasets for our analysis of network intrusion detection. Table 1 summarizes the overall scope of each dataset, including the number of features, the number of labels, and the number of samples.

### 3.2. Machine Learning Model

We trained and assessed four black-box classifiers that are commonly used in the IDS community.

RF: The Random Forest classifier was implemented using Scikit-learn with a fixed set of hyperparameters across all three datasets to maintain a controlled comparative setting. Each model used 100 decision trees, a minimum of 5 samples per leaf, a maximum tree depth of 15, and a minimum of 10 samples required to split an internal node. These values were selected based on preliminary trials to provide a reasonable balance between model complexity and generalization. It is acknowledged that dataset-specific hyperparameter tuning could potentially improve predictive performance for individual datasets. However, the purpose of this study is not to optimize each classifier separately but to evaluate XAI behavior under a consistent experimental protocol. Therefore, the same Random Forest configuration was retained across datasets to reduce the influence of tuning-related variability and support fairer cross-dataset comparison.

DNN: The architecture used in this research utilizes the Keras Sequential API and is designed to classify intrusion detection datasets. The architecture of this classifier receives as input a feature vector whose length matches the number of features in the dataset and feeds it into an initial input dense layer with 128 neurons using the rectified linear unit (ReLU) activation function, which is used for all datasets. Then there is a larger dense hidden layer with 256 neurons (with ReLU activation) that also uses L2 regularization (l2(0.001)) to penalize large weights and control overfitting. A dropout layer follows, in which 10% of the neurons were randomly dropped out in training to enhance generalization. Next, the model contains another dense layer of 64 neurons to continue the feature learning process, which also utilizes L2 regularization. The output dense layer contains several neurons equal to the number of classes for each dataset and a softmax activation function, which provides a probability distribution over target classes. The loss function was configured to use “categorical crossentropy,” while the adaptive momentum Adam was chosen as the optimization algorithm [42].

CNN: The classification model employed in this study is based on a convolutional neural network implemented using Keras’ Sequential API. The network begins with a Reshape layer that converts the input feature vector into a 4D tensor of shape (features, 1, 1) to enable compatibility with convolutional layers. It is important to note that the input data in this study is tabular and does not possess an inherent spatial structure. Therefore, the use of convolutional layers is not intended to model spatial locality but rather to provide a learnable transformation over the feature space. A Conv2D layer with 64 filters and a kernel size of (1,1) is then applied with ReLU activation. This operation can be interpreted as a feature-wise transformation that enables interaction across input dimensions without imposing artificial spatial assumptions. The output is flattened into a one-dimensional vector using a Flatten layer. Subsequently, a fully connected dense layer with 32 units and ReLU activation is used to extract higher-level feature representations. Finally, a dense output layer with a number of units equal to the number of classes and a softmax activation function produces the class probability distribution. The adopted CNN architecture is used as a lightweight and consistent backbone within the proposed evaluation framework, rather than as an optimized architecture for tabular data.

LSTM: The classification model employed in this study is based on a Long Short-Term Memory (LSTM) network implemented using Keras’ Sequential API. The model receives the tabular input in the form of an ordered feature sequence with shape $\left(n_{\mathrm{feats}},1\right)$, where $n_{\mathrm{feats}}$ denotes the number of input features. It is important to note that the tabular features do not inherently represent a natural temporal sequence. Therefore, the LSTM architecture is not used here to model true temporal dependencies but rather as a recurrent feature interaction model that processes the ordered feature vector sequentially to learn dependencies across feature dimensions. The network begins with an LSTM layer, followed by a stack of fully connected dense layers with 20, 60, 80, and 90 units, respectively. These layers are used to progressively transform the learned hidden representation into higher-level discriminative features. The final output layer is a dense layer with a number of units equal to the number of classes in each dataset, using a softmax activation function to produce a probability distribution over the target classes. The adopted LSTM architecture is used as a comparative backbone within the proposed evaluation framework, rather than as a claim that the original tabular feature ordering possesses intrinsic sequential semantics.

### 3.3. XAI Methods

Two post hoc explanation methods were employed in this study: LIME and SHAP. These methods were used to generate feature-attribution scores for intrusion detection samples and to support the evaluation of sparsity, completeness, and robustness under adversarial perturbation.

LIME: LIME was implemented using LimeTabularExplainer, with the training data used as the reference distribution for perturbation sampling. The explainer was configured in classification mode, with feature names defined according to the input columns and class names specified according to the target labels. For each evaluated sample, LIME generated perturbed instances around the input and fitted an interpretable surrogate model to approximate the classifier behavior in the neighborhood of that sample, yielding feature weights that reflect the contribution of individual input variables to the prediction [6].

SHAP: SHAP explanations were computed using KernelExplainer, which estimates additive feature-attribution scores based on Shapley values from cooperative game theory [17]. To construct the background reference distribution, the training data were summarized using shap.kmeans with 10 representative clusters. This summarized background set was then used during SHAP value estimation for each evaluated sample, and the resulting SHAP scores were used to quantify the contribution of individual features to the model prediction.

SHAP and LIME were selected because they are among the most widely used post hoc explanation methods in explainable AI and intrusion detection research [7,13,14]. In addition, they represent two distinct explanation paradigms: surrogate-based explanation in the case of LIME and additive feature attribution in the case of SHAP [6,17]. Their use in this study is therefore motivated by their methodological diversity and their relevance for comparative evaluation under adversarial perturbation, rather than by any prior assumption of reliability or trustworthiness. Compared with traditional techniques such as Leave-One-Column-Out (LOCO) [43], Individual Conditional Expectation (ICE) [44], and Partial Dependence Plots (PDP) [45], SHAP and LIME provide instance-level feature-attribution scores, which enable a more detailed analysis of model behavior in adversarial intrusion detection settings.

### 3.4. XAI Evaluation Metrics

The quality of explanations is evaluated using three primary metrics: sparsity, completeness, and robustness. Each metric offers a different perspective on how reliable and interpretable the explanations are under adversarial perturbations. Next, we describe each metric in detail and how it is measured within our evaluation framework.

Sparsity: We assess the degree of explanation sparsity for each AI model using Cumulative Thresholded Sparsity (CTS) curves derived from LIME and SHAP across the three datasets. The CTS curve characterizes how feature-importance scores are distributed after normalization to a common scale. At a given threshold value, CTS is defined as the fraction of features whose normalized importance values are less than or equal to that threshold. In this way, the CTS curve represents the cumulative distribution of feature-importance scores.

A sparse explanation is expected to assign low importance to most features while concentrating higher importance on only a small subset. Under this condition, the CTS curve rises rapidly at low threshold values, indicating that many features have relatively small importance scores. In contrast, when importance is distributed more evenly across a larger number of features, the CTS curve increases more gradually.

To summarize the overall behavior of the CTS curve, we compute the Area Under the Curve (CTS-AUC) using the trapezoidal rule. CTS-AUC is used here as a compact summary of explanation concentration across the full threshold range rather than at a single threshold value. Therefore, it provides a convenient comparative measure of sparsity across different XAI methods, models, and datasets. In this study, higher CTS-AUC values indicate more concentrated explanations, whereas lower CTS-AUC values indicate more diffuse importance distributions. We note, however, that CTS-AUC is intended to quantify explanation compactness rather than overall explanation quality by itself.

The computation is performed as follows:

1. Compute raw importance scores for all features of a given instance using LIME or SHAP.
2. Apply min-max normalization to map feature-importance values into the interval [0,1]. This normalization is introduced to place attribution scores from different explanation methods on a common scale, thereby enabling relative comparison of their concentration patterns. While this step may affect the absolute magnitude of the original scores, it is used here only to analyze the relative distribution of importance values across features.
3. Create a list of thresholds τ from 0 to 1 in increments of 0.1. For each threshold τ, count the number of normalized feature scores $S_{i}$ satisfying $S_{i}\leq \tau$.
4. At each threshold, compute the CTS score as
  (1) $$CTS\left(\tau \right)=\frac{\mathrm{number}\;\mathrm{of}\;\mathrm{features}\;S_{i}\leq \tau }{\mathrm{total}\;\mathrm{number}\;\mathrm{of}\;\mathrm{features}}.$$
5. Repeat Steps 3 and 4 for all threshold values and plot the CTS curve, where the x-axis represents τ and the y-axis represents CTS (τ).
6. Compute CTS-AUC using the trapezoidal rule to obtain a single summary measure of the concentration behavior captured by the CTS curve.

Completeness: In this work, completeness is operationalized as the extent to which the top-k features identified by an XAI method align with input dimensions that are sensitive to adversarial perturbation. Rather than claiming formal causal completeness, the proposed measure provides an empirical feature-alignment criterion under DeepFool-based perturbations. The objective is to examine whether an explanation method is able to identify at least part of the input feature set associated with decision-sensitive changes in the model.

For a given input sample *x*, the evaluation procedure is defined as follows:

1. Compute the top-k most important features for the clean input *x* using SHAP or LIME, and denote their indices by $F_{k}^{\mathrm{orig}}\left(x\right)$.
2. Use the DeepFool algorithm to compute a minimal adversarial perturbation vector δ such that f(x+δ)≠f(x).
3. Define intermediate perturbation levels as
  (2) $$x_{\alpha }=x+\alpha \cdot \delta ,$$
  where α∈[0,1] in steps of 0.1. The interval [0,1] was selected to represent the progression from the original input (α=0) to the full DeepFool perturbation (α=1), while the step size of 0.1 provides a practical balance between perturbation resolution and computational cost.
4. At each perturbation level α, identify the set of input features whose values have changed with respect to the original input *x*. A feature is considered perturbed if its value differs from the original input by more than a small numerical tolerance ϵ. This set is denoted by $P_{x,\alpha }$.
5. For each scaled perturbation $x_{\alpha }$, determine whether the original top-k explanation features overlap with the perturbed feature set, i.e.,
  (3) $$F_{k}^{\mathrm{orig}}\left(x\right)\cap P_{x,\alpha }\neq \emptyset .$$
6. Repeat this process across multiple samples for each attack class and compute the proportion of samples for which an overlap exists at each perturbation level.

The completeness score is then defined as

(4) $${\mathrm{COMP}}_{k}\left(\alpha \right)=\frac{1}{N}\sum_{i=1}^{N}1\left\{\exists f\in F_{k}^{\mathrm{orig}}\left(x_{i}\right)\;s.t.\;f\in P_{x_{i},\alpha }\right\},$$

this score measures the proportion of samples for which at least one of the original top-k explanation features overlaps with the perturbed feature set at perturbation level α. Here, $F_{k}^{\mathrm{orig}}\left(x_{i}\right)$ denotes the top-k features identified in the explanation of the original sample $x_{i}$, $P_{x_{i},\alpha }$ denotes the set of perturbed features at perturbation level α, and *N* is the total number of evaluated samples. This score should be interpreted as an alignment-based measure. It captures whether an explanation identifies at least one perturbation-sensitive feature at a given perturbation level. Although the formulation is intentionally binary and therefore coarse, the completeness curves evaluated across multiple α values provide a progressive view of explanation behavior under adversarial stress. Although completeness is evaluated under the same DeepFool-based perturbation framework used for robustness, it captures a different aspect of explanation behavior. Specifically, completeness measures whether the original top-k explanation features align with perturbation-sensitive input dimensions associated with changes in the model decision boundary. Therefore, completeness is interpreted as a feature-alignment property rather than an explanation-stability property.

Robustness: Robustness in this work is defined as the degree to which an explanation remains stable when the input is subjected to adversarial perturbation. Unlike completeness, which evaluates alignment with perturbation-sensitive input dimensions, robustness focuses on whether the explanatory feature rankings are preserved before and after the application of DeepFool.

For a given input sample *x*, we compute the top-k most important features before perturbation, denoted by $F_{k}^{\mathrm{orig}}\left(x\right)$, and after perturbation, denoted by $F_{k}^{\mathrm{adv}}\left(x_{\alpha }\right)$. The robustness score is then evaluated based on the overlap between these two sets across perturbation levels. A higher robustness score indicates that the explanation remains consistent under adversarial perturbation, while a lower score reflects instability in feature attribution. It is important to note that, although both completeness and robustness are evaluated using the same perturbation mechanism, they capture complementary properties of explanation quality. Completeness measures feature–alignment with perturbation-sensitive dimensions, whereas robustness measures the stability of explanations under adversarial stress.

Test-1 Robustness without Injected Feature Bias: In this evaluation, we investigate the robustness of SHAP explanations when the model is subjected to minimal adversarial perturbations. For each clean test instance, we compute SHAP values for the predicted class and select the top-k most important features, which act as the baseline explanation. We then apply the DeepFool algorithm to generate adversarial examples with minimal perturbations that change the decision of the model. We again apply SHAP to these adversarial examples to generate a new ranking of top-k features. Finally, we compare the explanations for clean and adversarial inputs to analyze how well the top features are preserved under an adversarial perturbation. Mathematically, let $f:R^{n}\to R^{C}$ be the trained classifier, and $\hat{Y}=\arg\max_{c}f_{c}\left(x\right)$ be the predicted class for input *x*. The SHAP attribution vector for *x* with respect to $\hat{Y}$ is:
  (5) $$\phi^{\mathrm{orig}}\left(x\right)=\left[\phi_{1}^{\mathrm{orig}},\phi_{2}^{\mathrm{orig}},\ldots ,\phi_{n}^{\mathrm{orig}}\right].$$

The minimal adversarial perturbation δ is obtained from DeepFool by solving the following:

(6) $$\delta =\arg\min_{\delta^{\prime }}{∥\delta^{\prime }∥}_{2}\;s.t.\;\arg\max_{c}f_{c}\left(x+\delta^{\prime }\right)\neq \hat{Y}.$$

The adversarial sample is: $x^{\mathrm{adv}}=x+\delta$. The SHAP attribution vector after attack is:

(7) $$\phi^{\mathrm{adv}}\left(x\right)=\left[\phi_{1}^{\mathrm{adv}},\phi_{2}^{\mathrm{adv}},\ldots ,\phi_{n}^{\mathrm{adv}}\right].$$

Then, we perform a qualitative or list-based comparison between $F_{k}^{\mathrm{orig}}\left(x\right)$ and $F_{k}^{\mathrm{adv}}\left(x\right)$ to evaluate attribution stability under adversarial perturbation:

(8) $$F_{k}^{\mathrm{orig}}\left(x\right)=\mathrm{indices}\;\mathrm{of}\;\mathrm{top}\text{-}k\;\mathrm{features}\;\mathrm{in}\;\left|\phi^{\mathrm{orig}}\left(x\right)\right|.$$

(9) $$F_{k}^{\mathrm{adv}}\left(x\right)=\mathrm{indices}\;\mathrm{of}\;\mathrm{top}\text{-}k\;\mathrm{features}\;\mathrm{in}\;\left|\phi^{\mathrm{adv}}\left(x\right)\right|.$$

Note that Test-1 is designed as a SHAP-specific robustness analysis rather than a direct comparative evaluation. The direct comparison between SHAP and LIME is conducted in Test-2 and Test-3.

Test-2 Robustness under Biased Feature Influence: In this evaluation, we examine how XAI methods behave when the model is trained with a deliberately injected spurious feature. Specifically, we introduced a synthetic, unrelated feature *u* into the dataset before training. This allows the model to assign spurious importance to the injected feature *u*. For each clean test instance, we compute SHAP or LIME values to obtain the baseline top-k feature ranks. We then apply the DeepFool algorithm to generate adversarial perturbations of the clean test instances and subsequently recompute the top-k features using SHAP and LIME on the perturbed inputs. By comparing the ranks of the original test instances and perturbed test instances, we determine whether the spurious feature became the dominant feature in the explanation under adversarial perturbations, therefore demonstrating a robustness failure.

Mathematically, let the feature set be $\{x_{1},x_{2},\ldots ,x_{n},u\},$ where *u* is the injected unrelated feature. SHAP/LIME attribution vectors

(10) $$\phi^{\mathrm{orig}}\left(x\right)=\left[\phi_{1}^{\mathrm{orig}},\phi_{2}^{\mathrm{orig}},\ldots ,\phi_{n}^{\mathrm{orig}},\phi_{u}^{\mathrm{orig}}\right],$$

(11) $$\phi^{\mathrm{adv}}\left(x\right)=\left[\phi_{1}^{\mathrm{adv}},\phi_{2}^{\mathrm{adv}},\ldots ,\phi_{n}^{\mathrm{adv}},\phi_{u}^{\mathrm{adv}}\right],$$

and top-k feature sets: $F_{k}^{\mathrm{orig}}\left(x\right),F_{k}^{\mathrm{adv}}\left(x\right)$. Robustness under bias is compromised if: $u\in F_{k}^{\mathrm{adv}}\left(x\right)$ and $u\notin F_{k}^{\mathrm{orig}}\left(x\right)$, showing that the spurious feature becomes overly dominant due to adversarial perturbation.

Test-3 Top-k Feature Overlap: The third robustness evaluation measures the consistency of feature attributions produced by SHAP and LIME before and after adversarial perturbation. Specifically, we assess whether each explanation method can maintain stable top-k feature rankings when subjected to input-level attacks designed to induce minimal decision-changing perturbations. For each dataset (BoT-IoT, Edge-IIoT, and N-BaIoT), we randomly selected 100 test samples. We applied SHAP and LIME to each original sample to extract the top 10 most important features. The input sample was then perturbed using the DeepFool algorithm, which generates minimal adversarial changes required to cross the model’s decision boundary. SHAP and LIME were again applied to the adversarial version of each sample to retrieve a new top-10 feature list. We computed the Top-k overlap ratio for each sample as the number of features common to both original and adversarial top-10 sets, divided by 10. This overlap ratio ranges from 0 (wholly distinct explanations) to 1 (same attributions). The average overlap over all 100 samples was calculated for each method and dataset. We also computed the 95% confidence interval for the mean overlap scores to assess the stability and uncertainty of the estimated robustness scores. Finally, we used paired *t*-tests and Wilcoxon signed-rank tests to quantitatively assess the robustness difference between SHAP and LIME. The paired *t*-test assumes that the differences between paired scores are approximately normally distributed, whereas the Wilcoxon signed-rank test does not require normality and is more appropriate when the distribution of differences is skewed or contains outliers. By applying both tests, we account for potential violations of the normality assumption and obtain a more reliable comparison of the two XAI methods. In this analysis, a higher mean overlap with narrower confidence limits indicates greater explanation stability under adversarial disturbance. Mathematically, let

$F_{k}^{\mathrm{orig}}\left(x\right)=\mathrm{top}\text{-}k\;\mathrm{features}\;\mathrm{for}\;\mathrm{clean}\;\mathrm{input}\;x$,

$F_{k}^{\mathrm{adv}}\left(x\right)=\mathrm{top}\text{-}k\;\mathrm{features}\;\mathrm{for}\;\mathrm{adversarial}\;\mathrm{input}\;x^{\mathrm{adv}}$. The overlap ratio is:

(12) $$R_{k}\left(x\right)=\frac{\left|F_{k}^{\mathrm{orig}}\left(x\right)\cap F_{k}^{\mathrm{adv}}\left(x\right)\right|}{k}.$$

The robustness score across a dataset of N samples is the mean overlap:

(13) $${\bar{R}}_{k}=\frac{1}{N}\sum_{i=1}^{N}R_{k}\left(x_{i}\right).$$

Then, compute the sample standard deviation:

(14) $$s=\sqrt{\frac{1}{N-1}\sum_{i=1}^{N}{\left(R_{k}\left(x_{i}\right)-{\bar{R}}_{k}\right)}^{2}}.$$

The 95% CI is: ${\mathrm{CI}}_{95}={\bar{R}}_{k}\pm t_{0.975,\;N-1}\frac{s}{\sqrt{N}}$, where $t_{0.975,\;N-1}$ is the critical value of Student’s *t* distribution with N-1 degrees of freedom. We compare SHAP vs. LIME robustness scores across the same N samples.

Let the differences be:

(15) $$d_{i}=R_{k}^{\mathrm{SHAP}}\left(x_{i}\right)-R_{k}^{\mathrm{LIME}}\left(x_{i}\right),\;i=1,2,\ldots ,N$$

compute the mean difference $\bar{d}=\frac{1}{N}\sum_{i=1}^{N}d_{i},$ and the standard deviation of differences: $s_{d}=\sqrt{\frac{1}{N-1}\sum_{i=1}^{N}{\left(d_{i}-\bar{d}\right)}^{2}}$. Assuming $d_{i}$ are approximately normal, we use the paired *t*-test and compute: $t=\frac{\bar{d}}{s_{d}/\sqrt{N}}$.

For the Wilcoxon signed-rank test, for each pair, compute the difference $d_{i}$, remove zero differences, and rank the absolute differences $\left|d_{i}\right|$ from smallest to largest, assign signs according to the original $d_{i}$, and compute the signed rank sums

$$W^{+}=\sum \mathrm{ranks}\;\mathrm{of}\;\mathrm{positive}\;d_{i},W^{-}=\sum \mathrm{ranks}\;\mathrm{of}\;\mathrm{negative}\;d_{i}$$

The test statistic is

(16) $$W=\min\left(W^{+},W^{-}\right).$$

In general, this framework evaluates robustness in a three-tier system.

Test-1: The qualitative stability of the explanation is assessed at the sample level through visual inspection to determine if the most salient features remain unchanged (or substantially different) between clean and adversarial explanations.

Test-2: Investigates how sensitive explanation methods are to the introduction of spurious features and whether they will highlight spurious features that contain no information when adversarial noise is added.

Test-3: Quantitatively determines the robustness of the explanation by calculating the overlap ratio (taking the top-k features) between clean and adversarial explanations across many samples, as well as calculating a numerical stability score that will be statistically validated using paired *t*-tests and Wilcoxon signed-rank tests.

### 3.5. DeepFool-Based Perturbation Protocol

Adversarial perturbations were generated using the DeepFool implementation provided by the Adversarial Robustness Toolbox (ART). The target classifier was wrapped using TensorFlowV2Classifier, with the number of output classes defined according to the dataset configuration and the loss function specified as categorical cross-entropy.

In our implementation, DeepFool was configured with a maximum of 100 iterations and an ϵ parameter of $10^{-2}$. The iterative attack procedure was applied until the classifier decision changed or the maximum iteration limit was reached. For an input sample *x*, DeepFool computes a minimal perturbation vector δ, and the corresponding adversarial sample is represented as $x^{adv}=x+\delta$.

To quantify the effectiveness of the DeepFool attack, we report the Attack Success Rate (ASR) using two complementary measures.

Let $x_{i}$ denote an input sample, ${\hat{y}}_{i}=\arg\max_{c}f_{c}\left(x_{i}\right)$ the predicted class before perturbation, and ${\hat{y}}_{i}^{adv}=\arg\max_{c}f_{c}\left(x_{i}^{adv}\right)$ the predicted class after perturbation. The overall attack success rate is defined as

(17) $${\mathrm{ASR}}_{all}=\frac{1}{N}\sum_{i=1}^{N}1\left({\hat{y}}_{i}^{adv}\neq {\hat{y}}_{i}\right),$$

where *N* is the total number of evaluated samples and 1(·) is the indicator function.

To account for the correctness of the original predictions, we also report the conditional attack success rate

(18) $${\mathrm{ASR}}_{cond}=\frac{1}{N_{c}}\sum_{i=1}^{N}1\left({\hat{y}}_{i}^{adv}\neq {\hat{y}}_{i}\wedge {\hat{y}}_{i}=y_{i}\right),$$

where $N_{c}$ denotes the number of samples that were correctly classified before the attack.

## 4. Results and Discussion

### 4.1. Results of Sparsity Metric

**Figure 2:**
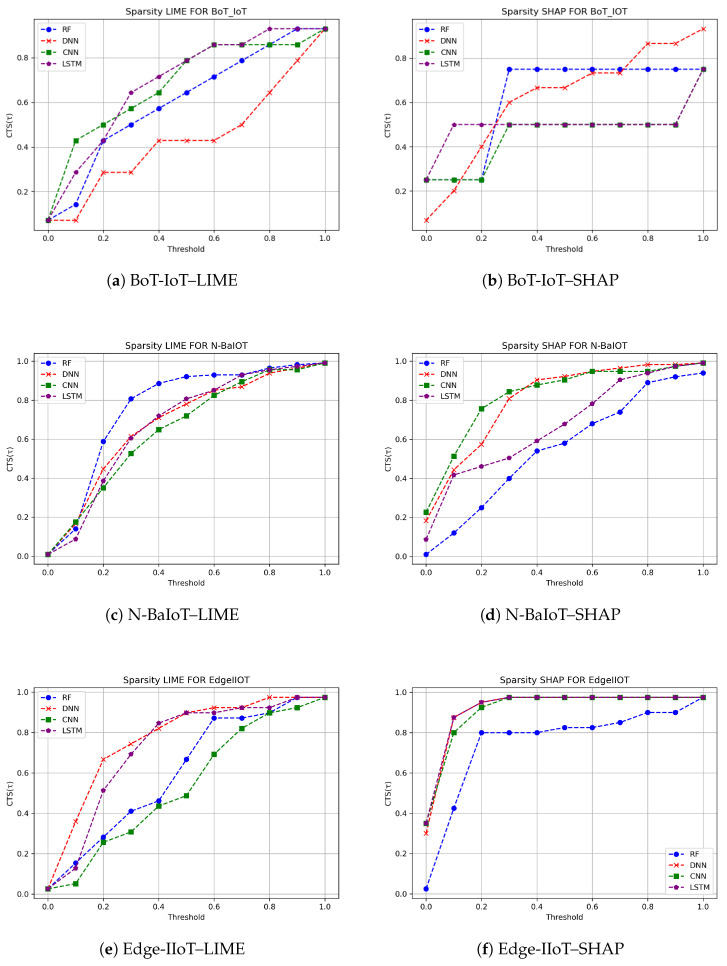
Sparsity results of the XAI methods across the three datasets. For each dataset, the left plot corresponds to LIME and the right plot corresponds to SHAP.

We present and discuss the sparsity results of SHAP and LIME explanations across the three IoT intrusion detection datasets: BoT-IoT, Edge-IIoT, and N-BaIoT. The corresponding Cumulative Threshold-Based Sparsity (CTS) curves are shown in Figure 2. These curves illustrate how the normalized feature-importance scores produced by SHAP and LIME are distributed across increasing threshold values (τ) from 0 to 1 in increments of 0.1 and can therefore be interpreted as cumulative distributions of explanation scores. A CTS curve that rises rapidly and remains close to one at relatively low threshold values indicates a more concentrated and therefore sparser explanation. In contrast, a curve that increases more gradually reflects a more diffuse distribution of feature importance across a larger number of features.

For the BoT-IoT dataset, the CTS curves in Figure 2a (LIME) and Figure 2b (SHAP) reveal clear differences in how sparsely each XAI method distributes importance across features. In Figure 2a, the LIME curves, especially for the CNN and LSTM models, rise rapidly and remain close to one, indicating very sparse explanations in which a small subset of features dominates the attribution and many potentially informative signals are largely ignored. In contrast, the SHAP curves in Figure 2b generally grow more gradually, reflecting explanations that are still relatively concentrated but spread importance over a broader set of features. These visual trends are supported by the CTS-AUC values in Table 2. For the DNN and RF models, SHAP attains slightly higher CTS-AUC values than LIME (0.623 vs. 0.436 for DNN and 0.625 vs. 0.607 for RF), indicating somewhat sparser yet still balanced explanations. For the CNN and LSTM models, however, LIME yields markedly higher CTS-AUC values than SHAP (0.671 vs. 0.450 for CNN and 0.693 vs. 0.500 for LSTM), confirming that LIME produces extremely sparse explanations that collapse onto a few dominant features. Overall, while both XAI methods are applied to highly accurate BoT-IoT classifiers, SHAP provides more balanced explanations over several relevant traffic features, whereas LIME, particularly for CNN and LSTM, tends to oversimplify the decision rationale by concentrating importance too narrowly.

For the N-BaIoT dataset, the CTS curves in Figure 2c,d show a more mixed sparsity pattern than in BoT-IoT. In Figure 2d, the SHAP curves for the DNN and CNN models rise rapidly and remain close to one over a wide range of thresholds, indicating highly concentrated explanations in which a small subset of features dominates the attribution. In contrast, the corresponding LIME curves in Figure 2c grow more gradually, suggesting less sparse, more diffuse explanations for these models. This behavior is consistent with the CTS-AUC values in Table 2, where SHAP achieves higher AUC than LIME for DNN (0.812 vs. 0.684) and CNN (0.832 vs. 0.655), reflecting more concentrated SHAP attributions in these two cases. For the RF model, the situation reverses: LIME attains a higher CTS-AUC than SHAP (0.765 vs. 0.559), indicating that LIME is more sparse and tends to collapse the explanation onto fewer features, while SHAP distributes importance more broadly. For LSTM, both methods exhibit similar CTS-AUC values (0.679 for SHAP vs. 0.682 for LIME), so neither shows a clear advantage in terms of sparsity. Overall, these results suggest that, on N-BaIoT, SHAP often provides concentrated yet structured explanations for DNN and CNN, whereas LIME can become overly sparse for RF, potentially overlooking subtler but still informative feature contributions.

For the Edge-IIoT dataset, the CTS curves in Figure 2e,f again highlight clear differences in how feature importance is distributed across models. In Figure 2e, the LIME curves show a noticeable rise at lower thresholds (between 0.1 and 0.3), particularly for the DNN and LSTM models, indicating that LIME concentrates part of its attribution on a subset of features while still assigning non-negligible importance to a wider set. By contrast, the SHAP curves in Figure 2f increase more sharply and remain close to one across a broader range of thresholds for all four models, especially CNN and RF, reflecting more strongly concentrated, highly sparse explanations in which a small group of features dominates the attribution. These visual patterns are confirmed by the CTS-AUC values in Table 2. SHAP consistently attains higher AUC values than LIME across all models—DNN (0.929 vs. 0.778), CNN (0.921 vs. 0.537), LSTM (0.931 vs. 0.729), and RF (0.763 vs. 0.609)—indicating that SHAP produces sparser explanations on Edge-IIoT systematically. While this strong sparsity enables SHAP to highlight a compact set of highly influential features, it also increases the risk of overlooking additional, more subtle signals that may contribute to the classifier’s decisions. In contrast, LIME yields less sparse, more diffuse explanations that distribute attribution more broadly, which may better reflect the complex structure of Edge-IIoT but at the cost of reduced conciseness and a higher cognitive load for analysts who must interpret a larger set of contributing features.

Taken together, the CTS curves and CTS-AUCs tell us a lot about how SHAP and LIME perform when used across all three types of IoT (BoT-IoT, N-BaIoT, and Edge-IIoT). In most setups (especially for DNN and CNN, and clearly on Edge-IIoT), SHAP produced much higher CTS-AUC than LIME; also, SHAP produced much steeper CTS curves than LIME. The resulting explanations from SHAP were mainly comprised a limited number of features that accounted for a disproportionate share of the reasoning behind SHAP’s predictions. SHAP is more useful for identifying important traffic indicators while possibly ignoring some of the nuanced signals that would be helpful in the decision-making process. Conversely, LIME had a much lower average CTS-AUC than SHAP, and LIME produced much flatter CTS curves than SHAP. Therefore, LIME’s explanations were typically made up of a significantly greater number of features than SHAP. However, on occasion, LIME’s explanations would become significantly sparse, with only a few dominant contributors to the prediction.

The overall outcome of this research is that SHAP can offer a relatively structured high-sparsity explanation that can allow use as a clearer indicator of feature importance, while LIME can provide a useful alternative in that it provides a more evenly distributed view of a broader number of contributors. The above findings confirm that predictive accuracy will not always correlate with the degree of explanation provided and that the sparsity and distribution characteristics of the XAI approach should be evaluated together with performance metrics of IoT intrusion detection.

### 4.2. Results of Completeness Metric

**Figure 3:**
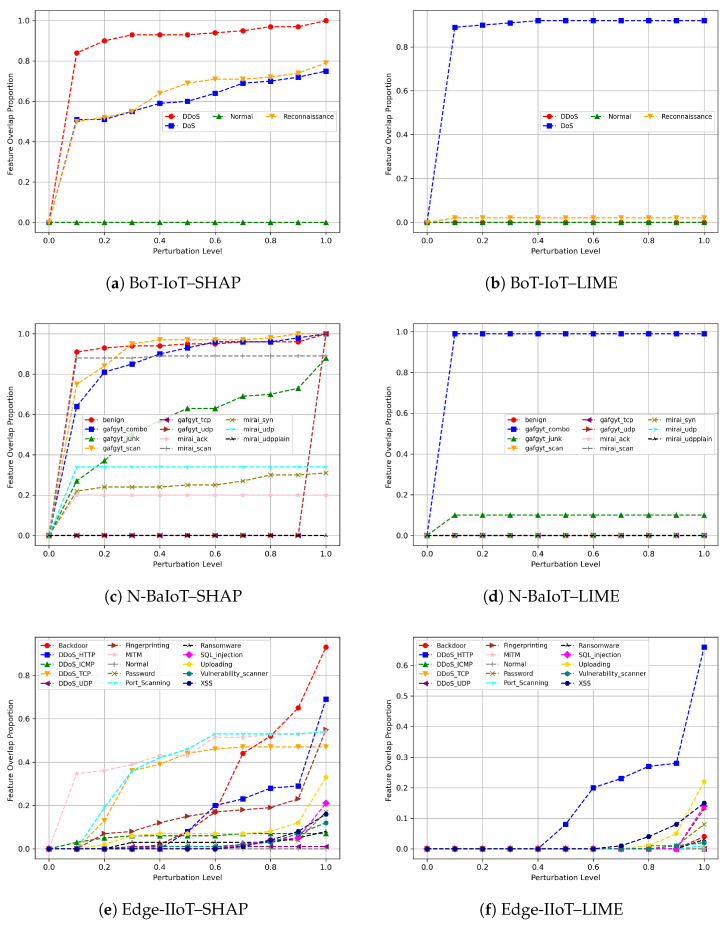
Completeness results of the XAI methods across the three datasets. For each dataset, the left plot corresponds to SHAP and the right plot corresponds to LIME.

Evaluating completeness in this work involves examining the extent to which the explanation methods, SHAP and LIME, align with perturbation-sensitive features under adversarial perturbation. Specifically, we analyze DeepFool-induced perturbations across three IDS datasets using 100 samples per dataset. The corresponding results are presented in Figure 3.

As shown in Figure 3a, SHAP exhibits higher feature-alignment completeness on the BoT-IoT dataset. As the DeepFool perturbation intensity (α) increases, the top-k SHAP features increasingly overlap with the perturbed feature set. This indicates that SHAP explanations remain more closely aligned with perturbation-sensitive input dimensions as the attack strength increases. In contrast, LIME shows noticeably lower completeness on the BoT-IoT dataset, as illustrated in Figure 3b. Even as α increases, the top-k LIME features show limited overlap with the perturbed features. This suggests weaker alignment between LIME explanations and the model’s perturbation-sensitive behavior under adversarial stress.

For the N-BaIoT dataset, SHAP again shows relatively high completeness, as seen in Figure 3c. Although this dataset appears more sensitive to adversarial perturbation, SHAP maintains meaningful overlap with the perturbed feature set across different values of α. This suggests that SHAP explanations remain partially aligned with perturbation-sensitive dimensions even when the model becomes more unstable under attack. By contrast, Figure 3d shows that LIME exhibits weaker and less consistent overlap across perturbation levels. In particular, the top-k LIME features do not track the perturbed feature set as reliably, indicating a greater loss of feature-alignment completeness under adversarial perturbation.

For the Edge-IIoT dataset, SHAP also demonstrates relatively strong completeness, as shown in Figure 3e. Its top-k feature attributions increasingly overlap with the DeepFool-perturbed features as the perturbation intensity grows, indicating that SHAP explanations remain broadly aligned with the model’s decision-sensitive input dimensions. However, compared with N-BaIoT, the increase in completeness for Edge-IIoT is more gradual. In contrast, LIME, shown in Figure 3f, remains less consistent, with limited overlap at lower values of α and weaker alignment overall as perturbation strength increases.

Overall, the completeness results illustrate how strongly the explanations produced by SHAP and LIME align with the feature dimensions affected by DeepFool across the evaluated IDS datasets. As perturbation intensity increases from clean to fully perturbed samples, SHAP consistently shows a clearer and more stable increase in overlap, indicating stronger feature-alignment completeness under adversarial stress. LIME, on the other hand, shows lower and more variable overlap, suggesting greater sensitivity to perturbation and weaker preservation of explanation relevance under attack. These findings highlight meaningful differences in how post hoc explanation methods behave in adversarial intrusion detection settings.

### 4.3. Results of Robustness Metrics

**Figure 4:**
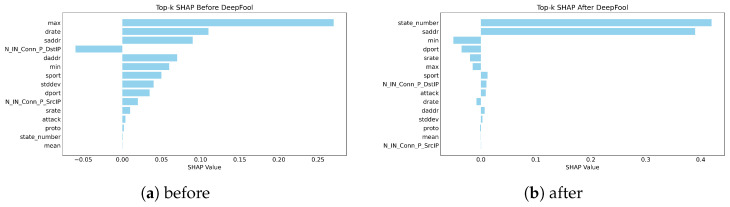
Test-1 robustness results of SHAP for the BoT-IoT dataset: (**a**) before and (**b**) after.

**Figure 5:**
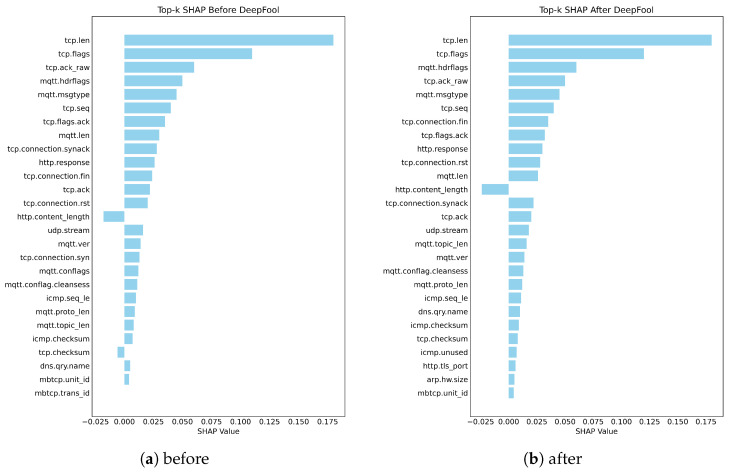
Test-1 robustness results of SHAP for the Edge-IIoT dataset: (**a**) before; (**b**) after.

**Figure 6:**
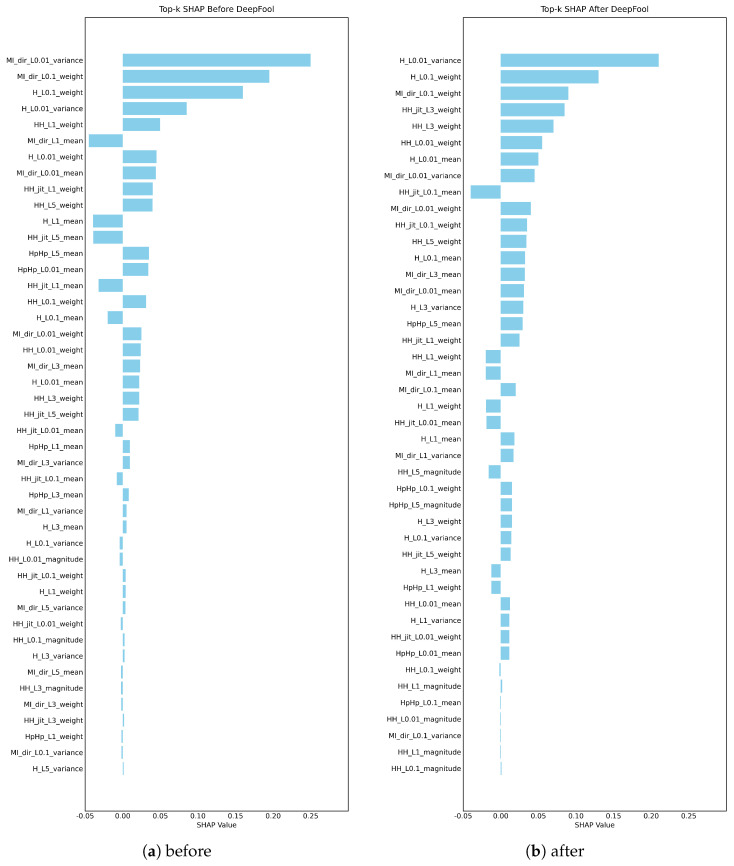
Test-1 robustness results of SHAP for the N-BaIoT dataset: (**a**) before and (**b**) after.

Test-1: We evaluate the robustness of SHAP under adversarial contexts with the DeepFool attack as our adversarial perturbation method before and after the application of the attack on the three analyzed datasets, as shown in Figure 4, Figure 5 and Figure 6.

Figure 4 shows SHAP explanations for the BoT-IoT dataset before and after being attacked by DeepFool, revealing that there was a considerable shift in feature attribution after the application of an adversarial attack. As illustrated in Figure 4a, the original SHAP explanation was composed mostly of small clusters of traffic-related variables that accounted for the largest portion of the model’s reasoning (i.e., maximum flow values, data rate, and number of inbound connections). After attacking the SHAP explanation using DeepFool, a new set of variables is present, as shown in Figure 4b, which comprises a collection of state and address descriptors, among other statistical variables. Many of the previously dominant features that were included in the original dataset have lost significance since their ranking decreased after being replaced with new features. These results show that the SHAP explanation is very sensitive to small perturbations in the input without any changes to either the architecture of the model or to how it was trained.

The SHAP explanations for the second dataset, Edge-IIoT, shown in Figure 5, show a significant alteration in the feature importance ranking due to the adversarial perturbation caused by DeepFool. As seen in Figure 5a, before the attack occurred, the most influential features pertained primarily to transport and application layers, including aspects of TCP segment length and flags, in addition to MQTT header data. Collectively, these factors represent domain-relevant stimuli, demonstrating that the model relies on behavioral characteristics at the protocol level to differentiate traffic patterns. However, after implementing the perturbation from DeepFool, as indicated in Figure 5b, a few of the protocol-related features remain among the highest ranked; however, the rankings and importance of those protocol variables noticeably change. In fact, many of the application-layer attributes previously deemed most influential lose significance; conversely, several features in the transport layer and connection-oriented domains become more prominent in the SHAP explanations. Overall, the rank change in feature importance, combined with the predicted class remaining the same, illustrates that DeepFool can alter the internal decision process of the model, despite an apparent similarity of the broad set of influential features.

For Figure 6, the SHAP explanations on the N-BaIoT dataset show a moderate but meaningful change in feature importance after the DeepFool perturbation. In Figure 6a, the highest-ranked variables correspond to statistical descriptors of traffic behavior (e.g., measures related to variance and weighted frequency), indicating that the model relies strongly on structured, frequency-aware characteristics of IoT traffic for anomaly detection. In Figure 6b, some of these statistical descriptors remain among the top features, but their relative importance changes, and several previously lower-ranked descriptors move up in the ranking. Overall, this partial reshuffling suggests that, beyond merely flipping the model’s prediction, the underlying reasoning of the classifier is altered by the adversarial perturbation, thereby compromising the consistency of explanations.

**Figure 7:**
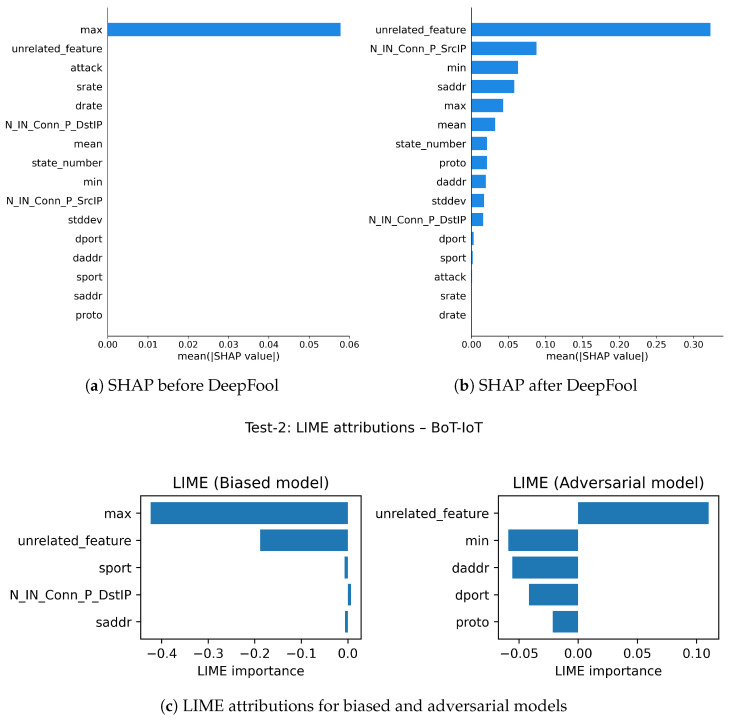
Test-2 robustness results for the BoT-IoT dataset: (**a**) SHAP before DeepFool; (**b**) SHAP after DeepFool; (**c**) LIME attributions for biased and adversarial models.

**Figure 8:**
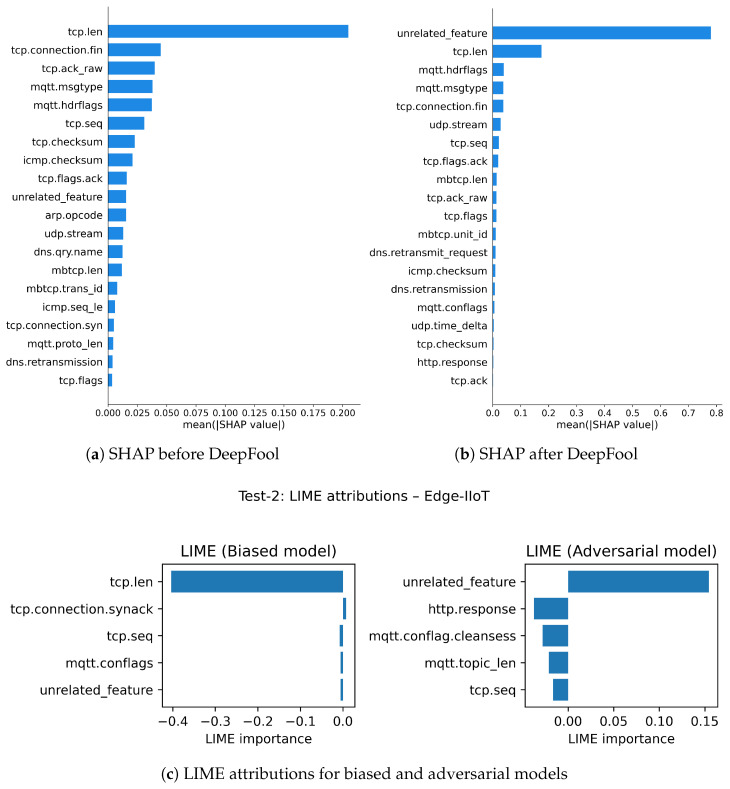
Test-2 robustness results for the Edge-IIoT dataset: (**a**) SHAP before DeepFool; (**b**) SHAP after DeepFool; (**c**) LIME attributions for biased and adversarial models.

**Figure 9:**
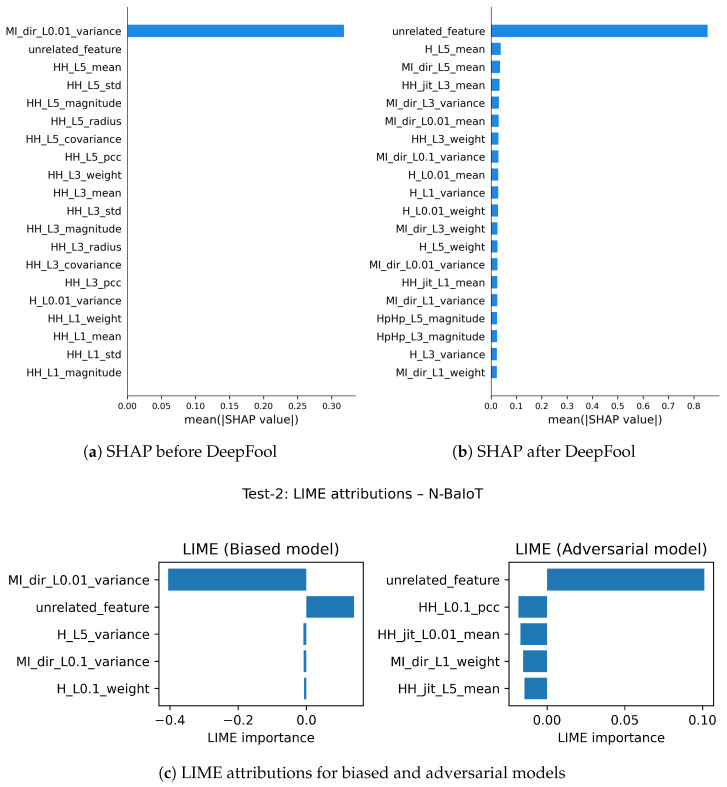
Test-2 robustness results for the N-BaIoT dataset: (**a**) SHAP before DeepFool; (**b**) SHAP after DeepFool; (**c**) LIME attributions for biased and adversarial models.

Test-2: With a fake unrelated feature, we evaluate the robustness of SHAP and LIME under adversarial contexts with the DeepFool attack as our adversarial perturbation method before and after applying the attack on three datasets, as shown in Figure 7, Figure 8 and Figure 9.

In the evaluation of robustness within the BoT-IoT dataset, SHAP maintains considerable stability and is more robust in its feature attributions when compared to LIME in the presence of adversarial perturbations (see Figure 7a,b). As discussed earlier, both methods initially identified the core features of the model completely; however, following perturbation using DeepFool, we see LIME’s explanations are altered extremely. In Figure 7c for LIME explanation, the injected synthetic feature appears at the top of the importance ranking, despite being explicitly designed as unrelated to the underlying classification task, which makes its prominence in the explanation difficult to justify. Certainly, SHAP does degrade in its explanation with perturbations, but not to the extent of LIME, which has a steadier loss in explanation fidelity as it still retains some of the original core features, modestly generating some recognizability under perturbation.

With the Edge-IIoT dataset, SHAP exhibited (see Figure 8a,b) the most overall robustness, maintaining valid explanations in the presence of adversarial perturbed inputs. When DeepFool was applied, SHAP rank changed only slightly while still emphasizing relevant features, while LIME’s explanations (see Figure 8c) prioritized the irrelevant feature. This suggests SHAP is more robust at maintaining causal signal integrity in structured and protocol-rich environments, such as Edge-IIoT. LIME’s explanations proved unstable and excessively sensitive to small perturbations and failed to reflect the model’s underlying logic under stress.

In the case of the N-BaIoT dataset, represented in Figure 9, the model is highly susceptible to DeepFool perturbations; minor perturbations can easily cause the model to make different predictions. Nonetheless, as shown in Figure 9a,b, SHAP explanations are somewhat more stable than LIME’s, retaining some of the original top features even after the model’s decision was subjected to an adversarial attack. In this specific instance, LIME’s explanations were much less coherent, as shown in Figure 9c, to the point where it even highlighted synthetic features that were entirely unrelated and irrelevant to the model’s decision-making process, demonstrating a breakdown in explanation integrity. Even though both explanation methods became weaker under the impact of significant attacks, SHAP was still more robust than LIME, so the user has some chance of obtaining partially useful explanations.

**Figure 10:**
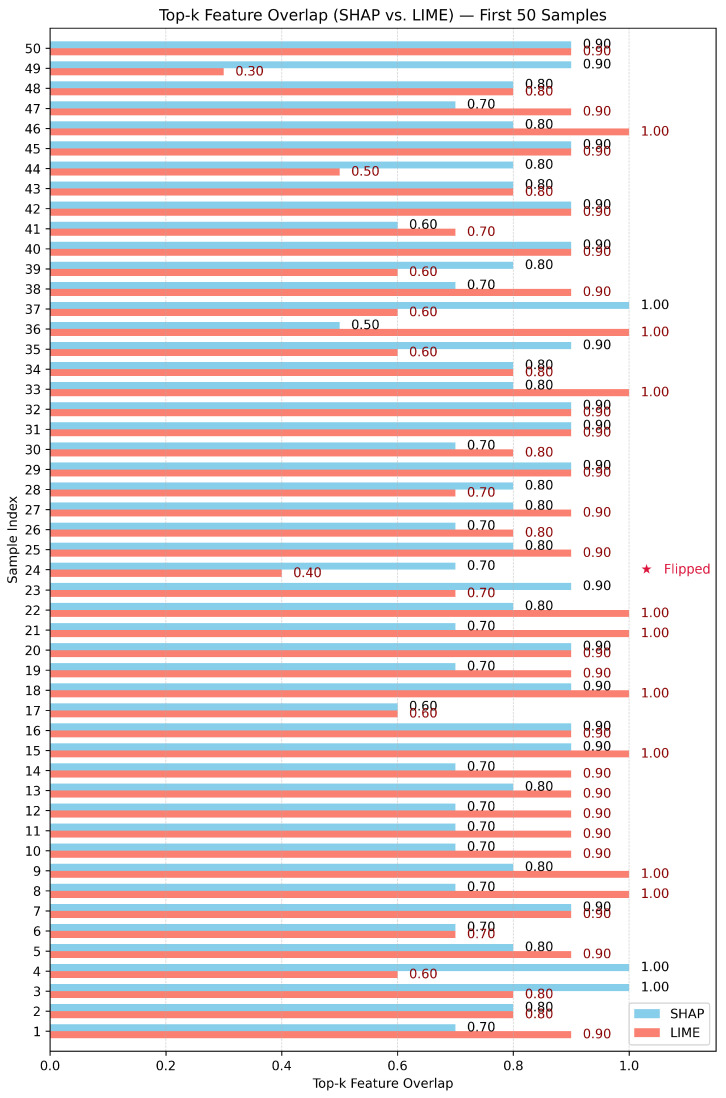
Test-3 robustness results of the XAI method for 50 samples of the Edge-IIoT dataset.

**Figure 11:**
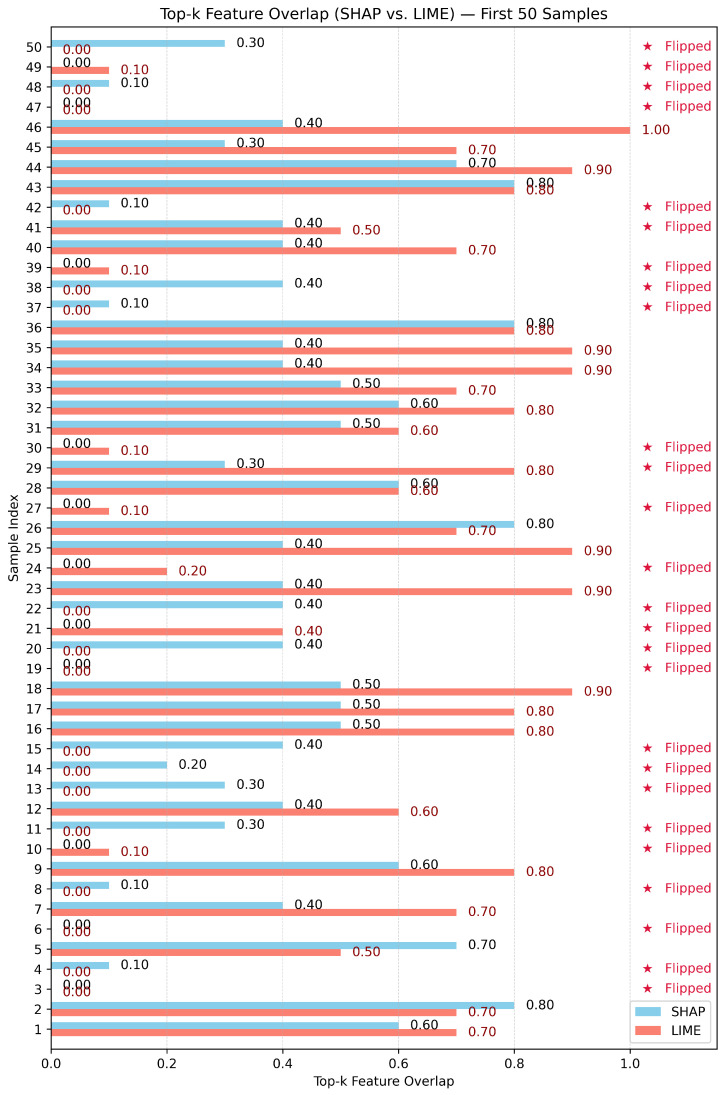
Test-3 robustness results of the XAI method for 50 samples of the N-BaIoT dataset.

**Figure 12:**
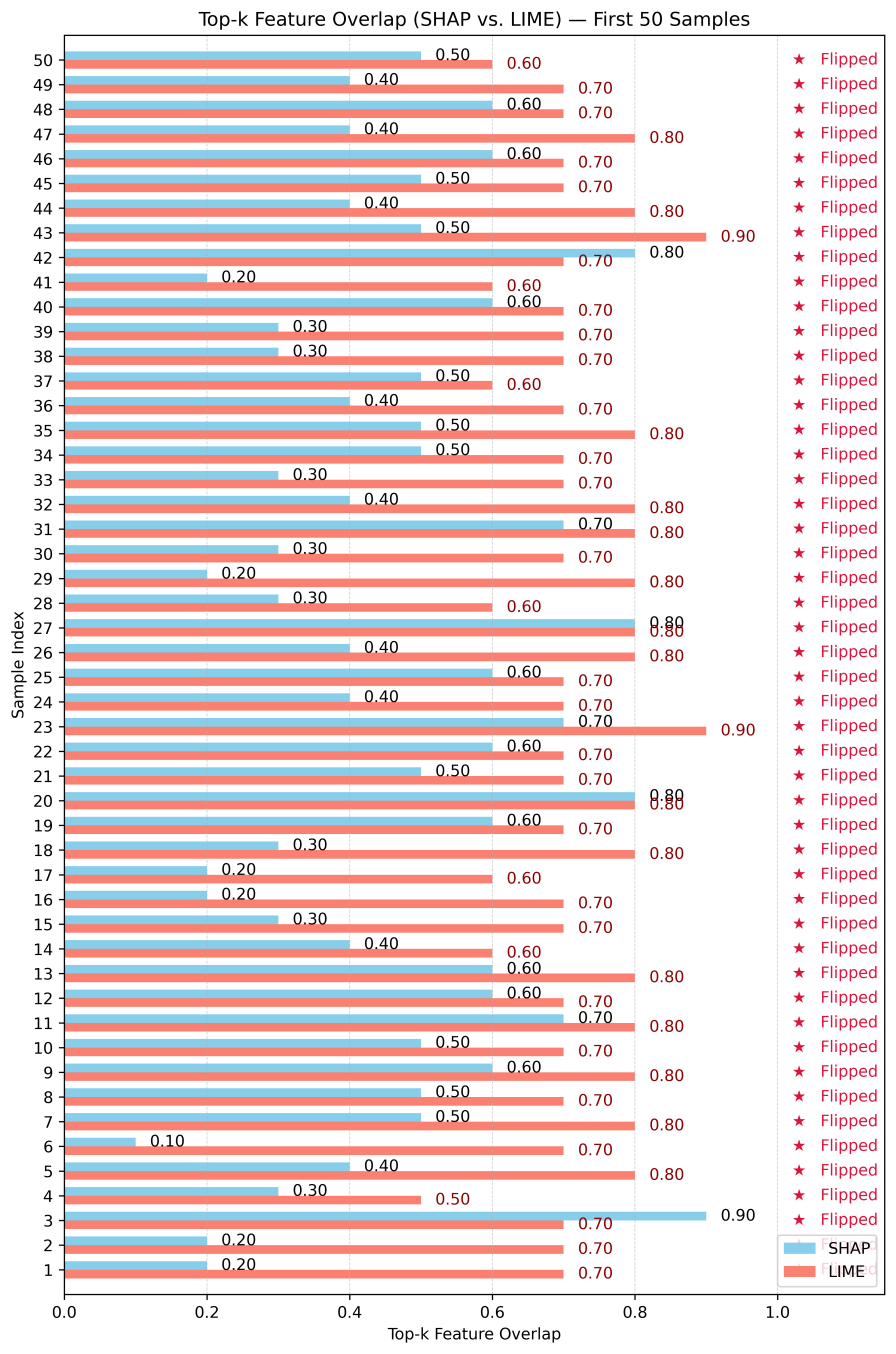
Test-3 robustness results of the XAI method for 50 samples of the BoT-IoT dataset.

Test-3: as shown in Figure 10, Figure 11 and Figure 12, this assessment utilizes the Top-10 Feature Overlap metric to quantify the robustness of SHAP and LIME under adversarial perturbations induced by the DeepFool algorithm. For each dataset, 100 test samples were evaluated. For each sample, SHAP and LIME were applied before and after perturbation to extract the top 10 most important features. The overlap ratio was calculated as the size of the intersection of the original and perturbed top-k sets divided by 10. A score of 1.0 indicates perfect consistency, while 0.0 means a complete shift in feature attribution.

With respect to the Edge-IIoT dataset, as shown in Figure 10, SHAP and LIME both exhibited high robustness to adversarial perturbations, with mean Top-k overlap scores reported as 0.799 (SHAP) and 0.851 (LIME) (Table 3). The stability advantage of LIME over SHAP was confirmed statistically (paired *t*-test: t = −2.746, *p* = 0.007; Wilcoxon: *p* = 0.006). In the cases where SHAP scores fell out of the high overlap scores (0.6–0.7), LIME remained consistently high (0.8 or above) for most samples. Class flips occurred at low frequency, and both methods preserved attribution reliability in the majority of instances.

In the case of the N-BaIoT dataset, as shown in Figure 11, both methods demonstrated decreased robustness overall. The SHAP method obtained a mean top-k overlap of 0.319 (95% CI: 0.272–0.366) versus 0.431 (95% CI: 0.354–0.508) for LIME, meaning the overall Top-k overlap associated with LIME was greater, but not particularly strong by comparison (Table 3). The difference between overlap accounts was statistically significant (paired *t*-test: t = −4.123, *p* < 0.001; Wilcoxon: *p* < 0.001), showing the feature attributions from LIME were more stable than those from SHAP in this dataset. However, evidence of class flipping and high variability across samples suggests a sensitive decision landscape that limits robustness for both explainers.

With the BoT-IoT dataset, the divergence in robustness, as shown in Figure 12, between methods was far more pronounced; while LIME had a high mean top-k overlap of 0.851 (95% CI: 0.821–0.881), SHAP generated a mean overlap of just 0.450 (95% CI: 0.415–0.485); a strong statistical result (paired *t*-test: t = −19.264, *p* < 0.001; Wilcoxon: *p* < 0.001) shows the overwhelming difference in stability. LIME was able to largely maintain its top-k set even across samples where prediction flips occurred, while SHAP’s attributions tended to be less stable under the same perturbations.

Throughout all three robustness tests, SHAP maintained less change than LIME in the face of adversarial perturbations that compromised the integrity of the explanations. In Test 2 (Robustness with unrelated feature), once again, SHAP demonstrated more resilience to resisting the rise of the synthetic, irrelevant feature to the top ranks, while LIME feature explanations were always susceptible and misguided. In Test 3 (Top-k Feature Overlap), however, LIME showed better robustness than SHAP for all three datasets by preserving a more consistent feature ranking for clean and adversarial inputs. LIME had higher mean Top-k overlaps, even when adversarial inputs flipped class labels (i.e., misclassifications), while attribution with SHAP declined sharply in stability. These results demonstrate an important trade-off: SHAP results in structurally grounded, model-aware explanations, but LIME showed greater robustness in the presence of adversarial stress and rank stability across feature ordering.

### 4.4. DeepFool Attack Effectiveness

Table 4 reports the effectiveness of the DeepFool attack across the evaluated datasets and classifiers. Overall, the results indicate that DeepFool successfully generates adversarial perturbations that frequently alter the model predictions across all experimental settings. Since DeepFool is a gradient-based attack, the attack success rate was computed only for differentiable classifiers (DNN, CNN, and LSTM). The Random Forest classifier was not included in this analysis because it does not provide the input gradients required by DeepFool.

For the Edge-IIoT dataset, all three differentiable models exhibit comparable vulnerability, with ${\mathrm{ASR}}_{all}$ values ranging from 0.840 to 0.856. This indicates that DeepFool consistently produces effective decision-changing perturbations for DNN, CNN, and LSTM under these datasets. Among the evaluated models, LSTM shows slightly higher susceptibility than DNN and CNN.

For the N-BaIoT dataset, the attack effectiveness varies more noticeably across classifiers. In particular, the LSTM model exhibits extremely high vulnerability, with ${\mathrm{ASR}}_{all}$ and ${\mathrm{ASR}}_{cond}$ values approaching 1.0, indicating that nearly all sampled inputs can be successfully perturbed. By contrast, the CNN model shows comparatively lower ASR values, suggesting relatively improved resistance under the same attack configuration.

For the BoT-IoT dataset, all evaluated models exhibit very high attack success rates, with ${\mathrm{ASR}}_{all}$ values exceeding 0.99. This indicates that DeepFool is highly effective in generating decision-changing perturbations for these datasets, despite the strong predictive performance of the classifiers. Among the evaluated models, the LSTM model reaches an ASR of 1.000, indicating complete vulnerability under the applied attack configuration, while DNN and CNN also show near-complete susceptibility.

Importantly, the close agreement between ${\mathrm{ASR}}_{all}$ and ${\mathrm{ASR}}_{cond}$ across all datasets indicates that the reported attack effectiveness is not driven only by already misclassified inputs. These results confirm that the explanation analyses in this work were conducted under genuinely effective adversarial perturbations, which strengthens the interpretation of the completeness and robustness evaluations.

## 5. Conclusions

This paper presents a unified empirical framework for examining the sparsity, completeness, and robustness of SHAP and LIME explanations for intrusion detection systems under DeepFool adversarial perturbations. Across three IoT datasets (BoT-IoT, Edge-IIoT, and N-BaIoT) and four classifiers, the results revealed clear trade-offs among these evaluation dimensions. In terms of sparsity, SHAP often produced higher CTS-AUC values and steeper CTS curves, indicating more concentrated explanations in which a smaller subset of features carried most of the attribution weight. By contrast, LIME generally produced less sparse explanations involving a broader set of contributing features, although in some cases it also yielded highly concentrated attribution patterns. Regarding completeness, SHAP consistently showed stronger feature-alignment completeness with DeepFool-perturbed input dimensions, indicating that its top-k features remained more closely aligned with perturbation-sensitive aspects of the model behavior than those of LIME. For robustness, SHAP showed stronger resistance to spurious feature influence in Test-2, whereas LIME achieved higher top-k overlap and greater ranking stability in Test-3, despite being more susceptible to synthetic or unrelated features.

Overall, the results suggest that explanation behavior cannot be inferred solely from model accuracy and should instead be examined from multiple complementary perspectives, including concentration, feature alignment, and stability under adversarial stress. For IDS practitioners, SHAP may be more suitable when stronger alignment with perturbation-sensitive model behavior is desired, whereas LIME may provide more stable feature rankings in some scenarios, albeit with greater sensitivity to spurious patterns.

Future work may extend this framework in several directions. First, additional IoT and IIoT datasets with diverse traffic characteristics can be incorporated to further examine the generalizability of the findings. Second, a broader range of explanation methods beyond SHAP and LIME can be investigated to provide a more comprehensive comparative analysis under adversarial perturbations. Third, alternative evaluation criteria may be developed to complement sparsity, completeness, and robustness, enabling a more complete assessment of explanation behavior. More broadly, this framework can also be extended to other security-critical domains, such as healthcare, finance, and autonomous systems. In addition, future research should explore methods for directly integrating robustness constraints into the explanation process.

## Figures and Tables

**Table 1:** Summary of the three network intrusion datasets used in this work.

Datasets	Number of Labels	Number of Features	Number of Samples
Edge-IIoT	15	63	2,219,201
N-BaIoT	11	115	1,854,174
BoT-IoT	4	19	3,668,521

**Table 2:** Quantitative results for the CTS metric.

Model	BoT-IoT		N-BaIoT		Edge-IIoT	
	SHAP	LIME	SHAP	LIME	SHAP	LIME
RF	0.625	0.607	0.559	0.764	0.762	0.608
DNN	0.623	0.435	0.811	0.684	0.928	0.778
CNN	0.450	0.671	0.832	0.655	0.921	0.537
LSTM	0.500	0.692	0.679	0.681	0.931	0.729

**Table 3:** Statistical comparison of SHAP vs. LIME results.

Dataset	SHAP Mean (95% CI)	LIME Mean (95% CI)	Paired *t*-Test (p)	Wilcoxon (p)
Edge-IIoT	0.799 (0.777, 0.821)	0.851 (0.821, 0.881)	0.007	0.006
N-BaIoT	0.319 (0.272, 0.366)	0.431 (0.354, 0.508)	0.000	0.000
BoT-IoT	0.450 (0.415, 0.485)	0.851 (0.821, 0.881)	0.000	0.000

**Table 4:** DeepFool attack success rates across datasets and classifiers.

Dataset	Model	${\mathrm{ASR}}_{\mathrm{all}}$	${\mathrm{ASR}}_{\mathrm{cond}}$
Edge-IIoT	DNN	0.848	0.834
Edge-IIoT	CNN	0.840	0.822
Edge-IIoT	LSTM	0.856	0.855
N-BaIoT	DNN	0.853	0.844
N-BaIoT	CNN	0.796	0.785
N-BaIoT	LSTM	0.994	0.995
BoT-IoT	DNN	0.991	0.991
BoT-IoT	CNN	0.994	0.994
BoT-IoT	LSTM	1.000	1.000

## Data Availability

The data supporting the reported results are publicly available as follows: (i) BoT-IoT dataset (UNSW Canberra Cyber): https://research.unsw.edu.au/projects/bot-iot-dataset (accessed on 14 January 2026); (ii) Edge-IIoTset dataset (IEEE DataPort): https://doi.org/10.21227/mbc1-1h68 (accessed on 14 January 2026); (iii) N-BaIoT dataset (UCI Machine Learning Repository): https://archive.ics.uci.edu/ml/datasets/detection_of_IoT_botnet_attacks_N_BaIoT (accessed on 14 January 2026). No new datasets were generated in this study.
